# Immediate public health surveillance response to cyclone Chido, Mayotte, 14 December 2024

**DOI:** 10.2807/1560-7917.ES.2025.30.6.2500060

**Published:** 2025-02-13

**Authors:** Marion Soler, Annabelle Lapostolle, Quiterie Mano, Maxime Ransay-Colle, Julie Durand, Louis Collet, Tanguy Cholin, Karima Madi, Laurent Filleul, Yvan Souares, Patrick Rolland, Hassani Youssouf, Response Group, The Response Group, Assiati Ahmed, Andani Andjilani, Fatima Ait El Belghiti, Caroline Alleaume, Mélissa Berot, André de Cafarelli, Laura Chaperon, Didier Che, Bruno Coignard, Marion Courtois, Duplexe Dammayao, Fatiha Djabour, Mathieu Epinoux, Julie Figoni, Erica Fougère, Nelly Fournet, Thierry Fouéré, Anne Fouillet, Loïc Grosse, Jérôme Guillevic, Anne Guinard, Jean-Paul Guthmann, Nassim Guy, Valérie Henry, François Herry, Alice Herteau, Guillaume Heuzé, Yasemin Inac, Asli Kilinc, Christine Larsen, Yann Le Strat, Andréa Linard, Philippe Malfait, Arnaud Mathieu, Mathilde Melin, Rahamata Moirabou, Anne Moulin, Harold Noël, Omdati Ousseni, Isabelle Parent du Chatelet, Damien Pognon, Jacques Rosine, Philippe Segura, Arnaud Tarantola, Mathieu Tourdjman, Aymeric Ung, Henriette de Valk, Michel Vernay, Ami Yamada

**Affiliations:** 1 Santé publique France Mayotte, Mamoudzou, France; 2 Santé publique France Provence-Alpes-Côte d'Azur, Marseille, France; 3 Agence Régionale de Santé de Mayotte, Mamoudzou, France; 4 Medical Biology Laboratory, Centre Hospitalier de Mayotte, Mamoudzou, France; 5 Santé publique France Nouvelle-Aquitaine, Bordeaux, France; 6 Santé publique France, Saint-Maurice, France; 7 The members of the Response Group are listed under Collaborators; * These authors contributed equally to this work and share first/last authorship.

**Keywords:** disaster, risk analysis, public health surveillance, vulnerable population, Mayotte

## Abstract

On 14 December 2024, Cyclone Chido caused extensive damage to infrastructures in Mayotte, including water, electricity and communication networks. Health surveillance systems were no longer functional. Santé publique France provided health risk analyses to support local control measures. Malnutrition and dehydration, follow-up care, pregnancy and post-partum complications, mental health, gastrointestinal diseases, bacterial wound superinfections and bronchiolitis were the main risk identified. The preliminary ad hoc surveillance systems confirmed our analyses. We also present lessons learnt 1 month after the event.

On 14 December 2024, the category 4 cyclone Chido reached the French island of Mayotte (Indian Ocean), with gusts of 250 km/h [1]. Chido destroyed the majority of households, and partially damaged the hospital and the four local medical centres. It also damaged the roads, the air traffic control, and shut down electricity, water and communications networks for a period between 3 days and more than 1 month, depending on the area. Sanitary conditions deteriorated and access to shelter, food, water and healthcare became limited. Health monitoring systems were no longer functional, including data from the hospital emergency department, hospitalisations, intensive care, networks of general practitioners and pharmacists, and vital statistics. Most laboratory-based surveillance remained operational. We here describe the immediate public health response led by Santé publique France, the national public health agency, defined to support control measures and countermeasures implemented by the local health authority. We also present preliminary results of the surveillance systems established on an ad hoc basis, and first lessons learnt 1 month after cyclone Chido.

## Demographic and socioeconomic overview of Mayotte

On 1 January 2024, the population of Mayotte was estimated at 321,000, based on follow-up adjustments since the latest comprehensive population census (2017) [2]. The population is younger than in mainland France (mean age: 23 vs 41 years). Between 2012 and 2017, average population growth was of + 3.8% per year, mainly driven by a surplus of births over deaths. In 2023, the fertility rate was well above the average for mainland France (4.5 children per woman vs 1.9). There are also sustained migratory movements, particularly from neighbouring Comoros mainly consisting of women aged between 15 and 34 and their children. Before cyclone Chido, 77% of Mayotte’s population lived below the poverty threshold and 40% of housing was informal [2,3]. Due to water shortage, access to running water was intermittent. In 2019, the obesity prevalence was 39.0% among women aged 15–69 years and 14.2% among men of the same age group [4]. This situation coexisted with malnutrition in children, with 7.1% being underweight (weight per height < −2 Z-scores) [4].

## Immediate public health response to cyclone Chido

The immediate response consisted of: (i) a risk assessment of communicable and non-communicable disease risks, based on literature and internal multidisciplinary expertise at Santé publique France, and (ii) establishing ad hoc public health surveillance systems.

### Communicable diseases risk assessment

A first risk assessment for communicable disease threats was issued on 15 December, relying on experiences with hurricanes in the Atlantic [5] and cyclones in the Indian Ocean [6], and based on a methodology adapted from the World Health Organization’s (WHO) risk assessment manual [7]. We prioritised the identified risks (Table 1) according to a risk score, calculated as the product of their likelihood of occurrence (scale 1 to 5) and potential consequences, i.e. impact on the population (scale 1 to 5).

**Table 1:** Prioritisation for communicable disease risks following cyclone Chido, Mayotte, France, 2024

Risk domains	Diseases	Likelihood of occurrence^a^ (1 to 5) [A]	Impact on the population^b^ (1 to 5) [B]	Risk score [A] × [B]
Faecal-oral infections	Gastrointestinal infections (incl. rotavirus)	4	4	**16**
	Cholera	2	4	8
	Typhoid fever	3	3	9
	Hepatitis A	3	2	6
Arbovirus infections	Dengue	1	4	4
	Chikungunya	2	3	6
	Rift valley fever	1	3	3
Cutaneous, eye and ENT infections	Scabies	3	3	9
	Conjunctivitis (bacterial and viral)	3	3	9
	Impetigo	3	3	9
	Bacterial superinfections of wounds	4	4	**16**
Soil-transmitted infections	Tetanus	2	3	6
	Melioidosis	2	3	6
Vaccine-preventable infections	Diphtheria	2	3	6
	Poliomyelitis	1	5	5
	Measles	2	4	8
	Invasive meningococcal disease	1	3	3
	Pertussis	1	3	3
Respiratory infections	Influenza	3	3	9
	COVID-19	2	2	4
	Bronchiolitis	4	3	**12**
	Tuberculosis	2	2	4
Others	Leptospirosis	3	3	9
	Malaria	1	3	3

ENT: ear, nose and throat.

^a^ Likelihood according to the epidemiological situation pre-existing (i) in Mayotte, (ii) in the region and (iii) internationally including hexagonal France.

^b^ Consequences for the population in terms of severity/extent and/or consequences for the healthcare system.

Scores [A] and [B] were assigned on a scale from 1 to 5 as follows: 1 = low, 2 = medium, 3 = high, 4 = very high, 5 = critical. Final risk scores higher than 10 are highlighted in bold.

Identified illnesses with the highest risk were faecal-oral diseases such as rotavirus infections, bacterial wound superinfections and acute respiratory infections already at epidemic and pre-epidemic stages before Chido such as, respectively, bronchiolitis and influenza. Other important risks were typhoid fever and leptospirosis (both endemic), mosquito-borne diseases (mainly chikungunya, possibly imported from the French island of La Réunion where the disease was circulating at the same time [8]), skin diseases and conjunctivitis, and vaccine-preventable diseases. Although the 2024 cholera outbreak in Mayotte has been over since July 2024 [9], it still circulated in the nearby Comoros in 2024 and was therefore still considered a substantial risk due to migration.

### Non-communicable diseases risk assessment

The non-communicable diseases health risks assessment was issued on 24 December, based on the same methodology (Table 2). We identified the highest non-communicable disease risks in the following areas: (i) malnutrition and dehydration: acute shortages of food and water, that already before Chido affected vulnerable populations where childhood malnutrition and maternal obesity coexist (double burden of malnutrition) [10,11]; (ii) post-cyclone accidental injuries: falls, wounds and haemorrhage associated with reconstruction activities, and intentional injuries linked with fights and assaults; (iii) deterioration of healthcare: treatment interruptions for chronic conditions such as renal failure, diabetes, hypertension and other chronic illnesses as well as pregnancy and post-partum complications, chronic asthma and newborn care; (iv) mental health issues: anxiety, depression and post-traumatic stress disorder, especially among displaced populations and emergency responders. These risks were exacerbated by ongoing food and social insecurity, and potentially related violence.

**Table 2:** Prioritisation for non-communicable disease risks following cyclone Chido, Mayotte, France, 2024

Risk domains	Health-related conditions or disease	Likelihood of occurrence^a^ (1 to 5) [A]	Impact on the population^b^ (1 to 5) [B]	Risk score [A] x [B]
Shortage of drinkable water	Dehydration	4	5	**20**
Food insecurity	Malnutrition	4	4	**16**
Unintentional injuries	Falls, wounds, haemorrhage	4	3	12
	Burns	2	2	4
	Animal bites and/or stings	1	1	1
	Drowning	1	1	1
Intentional injuries	Fights and assaults, including sexual assaults	3	4	12
Intoxications	Carbon monoxide poisoning	1	1	1
	Chemical poisoning	2	3	6
Deterioration of healthcare services	Chronic kidney disease – haemodialysis	3	5	15
	Chronic kidney disease – peritoneal dialysis	3	4	12
	Oxygen-dependent respiratory failure	2	4	8
	Pregnancy- and post-partum complications	4	4	**16**
	Essential newborn care	4	4	**16**
	Continuity of care for patients with chronic diseases	4	3	12
	Diabetes mellitus complications	4	4	**16**
	Cerebrovascular accident	3	3	9
	Myocardial infarctions	1	3	6
	Asthma	1	2	4
	(Local) healthcare access and follow-up care	5	5	**25**
Mental health	Acute stress, post-traumatic stress, burn-out	4	4	**16**

^a^ Likelihood according to the epidemiological situation pre-existing (i) in Mayotte, and (ii) in the region, and (iii) linked to post-cyclonic evolution.

^b ^Consequences for the population in terms of severity/extent and/or consequences for the healthcare system.

Final risk scores higher than 15 are highlighted in bold.

### Implementation of ad hoc public health surveillance systems

**Figure 1:**
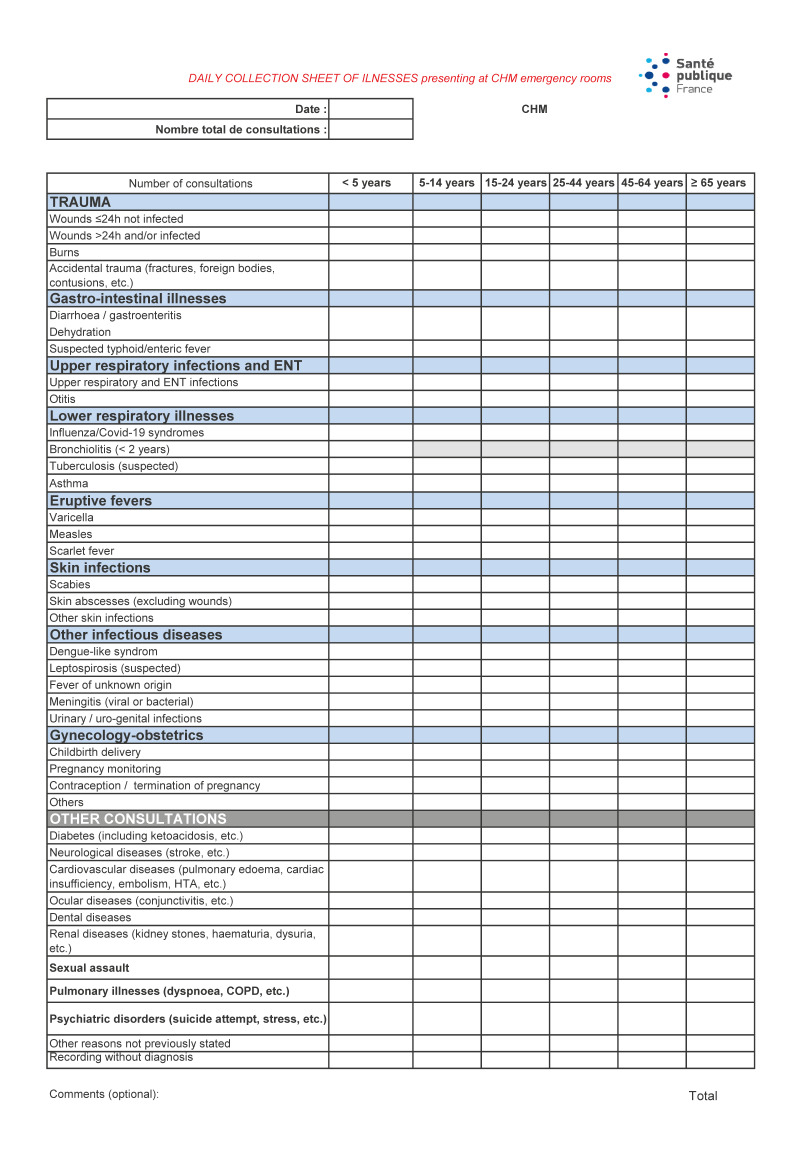
Data collection form used at Mayotte’s Hospital Emergency department, the field hospital, and local medical centres, after cyclone Chido, Mayotte December 2024

CHM: Centre hospitalier de Mayotte; COPD: chronic obstructive pulmonary disease; ENT: ear, nose and throat; HTA: hypertension.

Following the breakdown of surveillance systems affecting data collection and reporting, key challenges included lack of telephone and Internet networks, hindering communication with partners, transportation issues due to fuel shortages or damaged roads, and security concerns. To meet these challenges, Santé publique France established ad hoc health surveillance systems to monitor specific communicable and non-communicable disease risks among the general population. Within 48 h of cyclone Chido, the strategy focused on prioritising two streams of data collection: The first stream was devoted to data collection from the hospital emergency department and laboratory, from the four local medical centres and from the field hospital set up among other rescue operations (ESCRIM). The surveillance targeted nine categories of health conditions for six age groups, using paper forms (Figure 1) either photographed and transmitted via cell phone to ensure greater timeliness, or transmitted physically when the telecommunication network was unavailable. The second stream used the community-based surveillance system (CBS), first established in November 2023 during a water shortage crisis (unpublished data), in collaboration with health and social non-governmental organisations.

On 19 December 2024, CBS was strengthened to include epidemiologists for data collection together with healthcare professionals to provide first aid. Teams also distributed essential items such as chlorine tablets and soap, and educated on hygiene to help mitigate health risks. The CBS relies on syndromic data collected in children (up to 15 years) and adults, at the household level, on injuries, fever, diarrhoea, vomiting, cough and stress. It also enquires about the main sources of water supply. Local health mediators collected information while visiting neighbourhoods with poor living conditions to promote preventive actions (e.g. hygiene and nutrition) and alert health authorities to urgent cases. Documenting mortality remain a challenging issue, as official data are scarce, partly because vital statistics have since the cyclone not been transmitted from the town halls across the island. Alternative approaches to estimate cyclone-related mortality are under consideration [12].

## Preliminary results 

### Healthcare services and medical conditions

**Figure 2:**
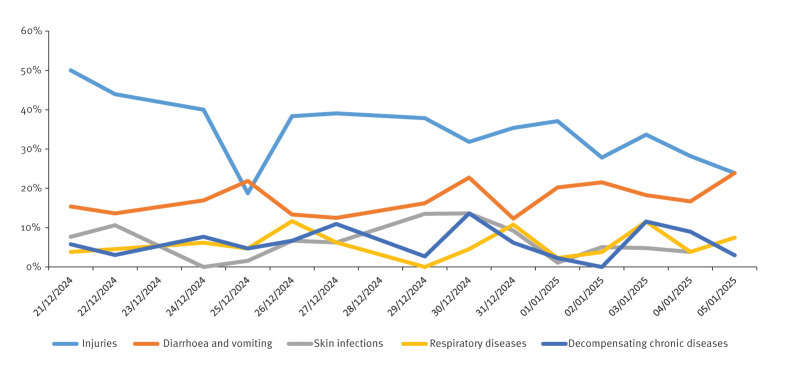
Main reasons notified^a^ for consulting Mayotte’s Hospital Emergency department, in percentage of total attendance^b^, Mayotte, 21 December 2024–5 January 2025

^a^ Data recorded daily between 7:00 and 20:00.

^b^ The indicators presented in this figure illustrate the reasons for consultation most frequently notified (one person may have had more than one condition) and those prioritised in our risk analyses. Therefore, the total does not add up to 100%.

From 14 December 2024 to 5 January 2025, the Mayotte hospital reported 4,043 emergency visits, primarily involving injuries (e.g. wounds, fractures) and gastro-intestinal disorders (Figure 2). Two of the four local medical centres reported similar cases. The field hospital, operational since 24 December 2024, provided care for 2,403 patients, with an average of 185 daily visits during the first week of January. A surge in gastro-intestinal disorders led to an overall positivity rate (PR) of stool samples greater than 80% for enteric pathogens during two consecutive weeks (W52–2024 to W01–2025), vs 60.8% and 68.3% for the two preceding weeks of 2024. Among the 134 samples tested in W01–2025, 111 were positive for at least one enteric pathogen. Specific PR of that magnitude had not been observed since November 2023 during the water crisis. Gastroenteritis, mostly associated with waterborne pathogens such as *Escherichia coli* (PR > 50%), *Shigella* (31%) and rotavirus (18%), were prominent among children younger than 2 years. Acute respiratory infections were also reported, including bronchiolitis considerably affecting children, and influenza. At the hospital, diabetic complications led to daily hospitalisations.

### Community-based surveillance

We visited 123 households during W52–2024, but the collected information was not of satisfactory quality. In our next round of outreach surveillance, we visited another 237 households during W01–2025. Among these 237 households across 14 neighbourhoods, 87 (37%) reported stress or psychological issues among adults and 78 (33%) among children. Twenty-eight (12%) reported physical injuries among children and 48 (20%) among adults, all linked to cyclone Chido. Fever and cough were prevalent, mostly affecting children. Gastroenteritis cases were reported in 32 (14%) of households among children and in 15 (6%) among adults. Malnutrition was diagnosed among children together with dehydration among breastfeeding mothers, worsened by uneven food distributions. Access to bottled water remained very low (< 10% of households). However, access to the drinkable water network increased from 36% in W52–2024 to 82% of households in W01–2025, while the consumption of untreated water decreased from 88% in W52–2024 to 38% of households. However, comparison remains limited, as different households were visited in the two weeks.

## Conclusion

Preliminary surveillance results showed that accidental injuries were the first reason to attend emergency care, followed by faecal-oral and skin diseases, decompensating chronic diseases and respiratory infections. These were consistent with our health risk analyses. The poor living conditions already existing before the cyclone have worsened. Delayed medical care resulted in excessive untreated injuries, disrupted management of chronic diseases and extensive psychological traumas. Inadequate access to clean water and latrines, combined with lack of soap, fostered the spread of waterborne diseases. All public health surveillance systems must now be strengthened to allow for a better assessment of the short-, medium- and long-term impact of cyclone Chido in terms of morbidity and mortality. Successive overwhelming crises since 2023, i.e. draught, cholera and Chido, highlight the need for adapting preparation and response plans in Mayotte.
